# Analgesic efficacy of ultrasound-guided ESPB on metabolic surgery

**DOI:** 10.3389/fmed.2025.1630657

**Published:** 2025-09-02

**Authors:** Ying Wang, Yan Liu, Wen Jie, Qiuya Yang, Fenglin Jiang

**Affiliations:** ^1^Department of Emergency, Clinical Medical College & Affiliated Hospital of Chengdu University, Chengdu, China; ^2^Department of Hospital Infection Management, Clinical Medical College & Affiliated Hospital of Chengdu University, Chengdu, China; ^3^Department of Nursing, Pengzhou Peoples’s Hospital, Pengzhou, China

**Keywords:** metabolic surgery, erector spinae plane block, regional anesthesia, meta-analysis, bariatric procedures

## Abstract

**Background:**

This systematic review and meta-analysis assessed the analgesic efficacy of ultrasound-guided ESPB in metabolic surgery.

**Methods:**

A systematic literature search of PubMed, the Cochrane Library, Web of Science, and Embase was conducted from database inception to February 2025 to identify randomized controlled trials (RCTs) comparing ultrasound-guided erector spinae plane block (ESPB) with either no block, sham block, or alternative regional analgesic techniques in patients undergoing metabolic surgery. Primary outcomes included 24 h postoperative opioid consumption, while secondary outcomes encompassed pain scores, time to first analgesic requirement, postoperative nausea and vomiting, and patient satisfaction. Risk of bias was assessed using the Cochrane Risk of Bias Tool, and evidence quality was evaluated using GRADE.

**Results:**

Ten RCTs involving 729 patients were included. ESPB significantly reduced 24 h opioid consumption [mean difference (MD): −6.68; 95% CI: −10.75, −2.61; *p* = 0.001] and resting pain scores at 24 h [MD: −0.78; 95% CI: −1.10, −0.46; *p* < 0.00001]. Movement pain scores were also reduced to 6, 12, 24, and 48 h (*p* < 0.00001 for all). ESPB prolonged the time to first rescue analgesic [MD: 14.17; 95% CI: 5.50, 22.85; *p* = 0.001]. However, no significant differences were observed in PONV incidence or patient satisfaction scores.

**Conclusion:**

Ultrasound-guided ESPB is an effective and safe analgesic technique for metabolic surgery, significantly reducing opioid consumption and pain scores while delaying the need for rescue analgesics.

**Systematic review registration:**

PROSPERO 2025 CRD420251000358. Available from https://www.crd.york.ac.uk/PROSPERO/view/CRD420251000358.

## Highlights

- Metabolic surgery is associated with considerable postoperative pain, which can impede recovery, delay ambulation, and increase the risk of pulmonary complications.
- Effective pain management is therefore critical to enhancing postoperative outcomes and patient satisfaction.
- Ultrasound-guided ESPB significantly reduces postoperative opioid consumption at 24 h compared to non-block care and sham block in patients undergoing metabolic surgery.
- ESPB prolonged the time to first rescue analgesic requirement, indicating its potential to reduce the need for additional opioid administration.

## 1 Introduction

Metabolic surgery, including bariatric procedures such as laparoscopic sleeve gastrectomy and Roux-en-Y gastric bypass, has become a cornerstone in the management of obesity and related metabolic disorders (1). While these surgeries offer significant health benefits, they are associated with considerable postoperative pain, which can impede recovery, delay ambulation, and increase the risk of pulmonary complications (2, 3). Effective pain management is therefore critical to enhancing postoperative outcomes and patient satisfaction (4).

Opioids have traditionally been the mainstay of postoperative analgesia, but their use is fraught with significant side effects, including respiratory depression, sedation, nausea, vomiting, and the potential for addiction (5). In the context of metabolic surgery, where patients often have comorbidities such as obstructive sleep apnea and obesity hypoventilation syndrome, the risks associated with opioid use are particularly pronounced (6, 7). Consequently, there is a growing interest in opioid-sparing analgesic strategies, with regional anesthesia techniques playing a pivotal role. The erector spinae plane block (ESPB) has gained attention as a novel regional anesthesia technique that provides effective analgesia for a variety of surgical procedures (8–10). By injecting local anesthetic into the fascial plane deep to the erector spinae muscle, ESPB can block the dorsal rami of spinal nerves, providing both somatic and visceral analgesia (11, 12). Its ease of performance under ultrasound guidance, combined with a favorable safety profile, makes ESPB an attractive option for postoperative pain management in metabolic surgery (13, 14).

Despite its potential, the efficacy of ESPB in metabolic surgery has not been comprehensively evaluated. Although a meta-analysis encompassing six studies, including both randomized controlled trials (RCTs) and observational cohort studies, demonstrated that bilateral ESPB offers opioid-sparing analgesia and superior pain scores compared to the control group, the limited sample size and heterogeneity of the included studies may compromise the overall quality of the research (2). Additionally, existing studies have reported conflicting results regarding its impact on opioid consumption, pain scores, and patient-reported outcomes such as quality of recovery and satisfaction. Furthermore, the comparative effectiveness of ESPB relative to other regional anesthesia techniques, such as the transversus abdominis plane block (TAPB) and quadratus lumborum block (QLB), remains unclear.

This systematic review and meta-analysis aimed to synthesize the available evidence from RCTs to evaluate the analgesic efficacy and safety of ultrasound-guided ESPB in patients undergoing metabolic surgery. By addressing these questions, we hope to provide clinicians with evidence-based recommendations for incorporating ESPB into multimodal analgesic regimens, ultimately improving postoperative outcomes in this high-risk patient population.

## 2 Methods

This study was conducted in accordance with the PRISMA (Preferred Reporting Items for Systematic Reviews and Meta-Analyses) Guidelines (15). The meta-analysis was prospectively registered in the PROSPERO database (CRD420251000358).

### 2.1 Search strategy

A comprehensive search was performed across multiple electronic databases, including PubMed, The Cochrane Library, Web of Science Citation Index, and Embase, from their inception to February 2025. The search aimed to identify randomized controlled trials (RCTs) meeting predefined inclusion criteria. Search terms were selected based on the PICOS framework and included: “laparoscopic bariatric surgery,” “bariatric surgery,” “metabolic surgery,” and “erector spinae plane block.” Additionally, reference lists of identified articles were screened to ensure thorough search. No language restrictions were applied during the search process. The detailed search strategy is provided in the Supplementary Digital Content. Grey literature was also searched through manual screening, focusing on ESPB-related studies, which were first introduced in 2016.

### 2.2 Study selection criteria

Two independent investigators conducted the literature search and screening. Disagreements were resolved through discussion with a third reviewer. Eligible studies included full-text RCTs comparing the analgesic efficacy of ESPB with non-block care or other blocks in adult patients undergoing metabolic surgery. Case reports, non-RCT studies, incomplete clinical trials, and conference abstracts lacking sufficient study design or data (even after contacting authors) were excluded. No language restrictions were applied during study selection.

### 2.3 Data extraction

Data extraction was performed systematically and included the following variables: authors, publication year, country, surgical procedure, use of ultrasound guidance, puncture location, type and dosage of local anesthetics, postoperative pain scores, postoperative pain management, opioid-related side effects (e.g., postoperative nausea and vomiting, PONV), and complications associated with ESPB (e.g., nerve damage, local anesthetic toxicity, pneumothorax, hematoma, or infection at the puncture site). Pain scores were assessed using either a visual analogue scale (VAS) or a numeric rating scale (NRS), which were standardized to a 0–10 scale for statistical analysis (0 = no pain, 10 = extreme pain).

The primary outcome was postoperative opioid consumption (measured in morphine equivalents) at 24 h. Secondary outcomes included age, BMI, postoperative pain scores at rest and during movement at various time points, duration of surgery and anesthesia, time to first analgesic requirement, time to first ambulation, length of hospital stay, PONV, patient satisfaction scores, quality of recovery15/40 (QoR15/40), and regional blocks related complications. For studies presenting data graphically, numerical data were extracted using WebPlot Digitizer (16). Perioperative opioid consumption was converted to intravenous morphine equivalents using a standardized conversion calculator (17).

### 2.4 Risk of bias assessment

Methodological quality assessment was independently assessed by two authors, with any disagreements resolved by a third author, according to the Cochrane Risk of Bias Tool 2 (each article was recorded either as low risk of bias, some concerns, or high risk of bias). Finally, the Grading of Recommendations Assessment, Development, and Evaluation (GRADE) methodology was performed to assess the quality of evidence for each outcome (18). Evidence quality was rated as low, moderate, or high based on outcome-specific and comparison-specific criteria. A flow chart was used to illustrate the study selection process and reasons for exclusion.

### 2.5 Statistical analysis

All statistical analyses were performed using Review Manager (version 5.4; The Nordic Cochrane Center, Copenhagen). Studies with similar outcome measures were included in the meta-analysis. Continuous data, such as postoperative pain scores and opioid consumption, were expressed as means and standard deviations (SD). Mean differences (MD) with 95% confidence intervals (CI) were calculated using a random-effects model. For studies reporting medians and interquartile ranges (IQR), these values were converted to means and SDs using the method described by Hozo et al. (19). Dichotomous data, such as PONV, were analyzed as relative risks (RRs) with 95% CIs using the Mantel–Haenszel method (20). To avoid redundant sample size assessments in multi-arm studies, the number of participants is evenly distributed. In cases where there are one intervention group and two control groups, the number of patients in the intervention group is proportionally allocated to enable comparisons with each of the control groups.

Heterogeneity was assessed using the I^2^ statistic. A random-effects model was applied if I^2^ > 50%, indicating substantial heterogeneity; otherwise, a fixed-effects model was used (20, 21). Sensitivity analyses were conducted using the leave-one-out approach to identify potential sources of heterogeneity for primary outcomes (postoperative 24 h resting pain score and opioid consumption). The methodological quality of individual studies was evaluated using the Cochrane Risk of Bias Tool 2 for RCTs (22), focusing on sequence generation, allocation concealment, blinding of participants and outcome assessors, incomplete data, and selective reporting.

## 3 Results

### 3.1 Results of literature search

**Figure 1:**
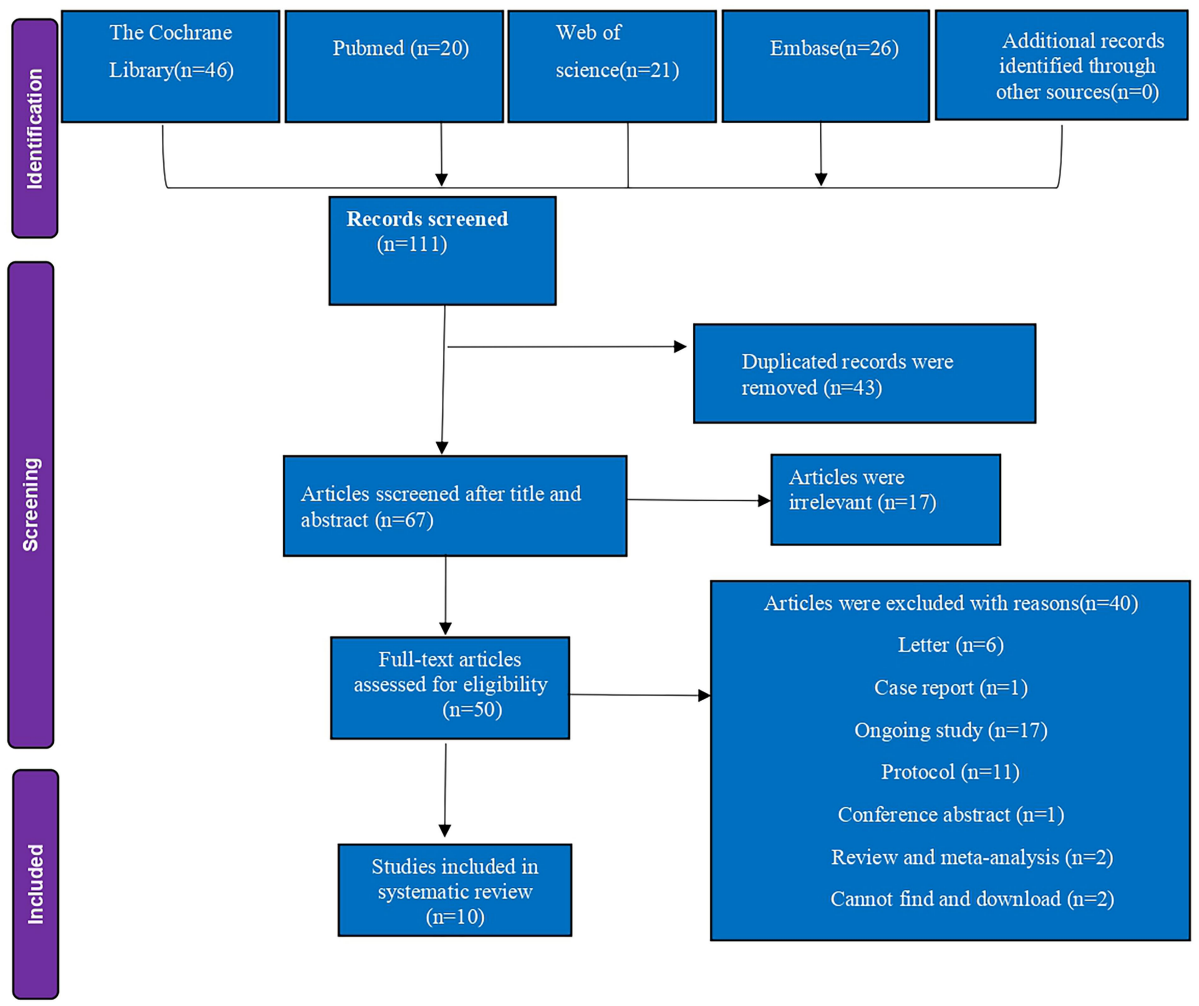
PRISMA flow diagram of included and excluded studies. PRISMA, preferred reporting items for systematic reviews and meta-analyses.

The study selection process is illustrated in the PRISMA flow diagram (Figure 1). The initial literature search identified 111 citations across four electronic databases. After full-text review and application of exclusion criteria, 10 RCTs involving 729 patients were included in the analysis (6, 7, 13, 14, 23–28). Table 1 summarizes the characteristics and details of these studies. All 10 articles were published between 2020 and 2025 and were written in English. The studies originated from Egypt (*n* = 4) (6, 7, 13, 14), Turkey (*n* = 3) (24, 25, 28), Thailand (*n* = 1) (23), Kingdom of Saudi Arabia (*n* = 1) (26), and China (*n* = 1) (27).

**Table 1:** Characteristics of included studies.

Study	Country	No. of patients (intervention vs. control)	Age (intervention vs. control)	Surgery	Intervention	Control arm	Primary outcome	Secondary outcomes	Conclusion
Abdelhamid., et al. 2020 (6)	Egypt	22 vs. 22 vs. 22	37.1 ± 10.4 vs. 35.9 ± 8.8 vs. 35.7 ± 8.6	Laparoscopic sleeve gastrectomy	US-guided bilateral ESPB	1. US-guided bilateral TAPB 2. Control	VAS pain scores	[1, 2, 3, 6]	ESPB lowers postoperative pain scores and reduces intraoperative and postoperative opioid consumption compared with both the subcostal approach TAPB and the control group.
Ashoor., et al. 2023 (7)	Egypt	32 vs. 34 vs.35	33.8 ± 5.4 vs. 34.3 ± 6.8 vs. 34.7 ± 6.7	Laparoscopic sleeve gastrectomy	US-guided bilateral ESPB	1. US-guided bilateral QLB 2. Control	The time to first rescue analgesia	[1, 2, 5]	Bilateral ESPB and QLB provided adequate postoperative pain control and reduced postoperative analgesic requirements for morbid obese patients.
Elshazly., et al. 2022 (13)	Egypt	30 vs. 30	35.37 ± 6.16 vs. 35.6 ± 6.37	Laparoscopic bariatric surgery	ESPB	TAPB	Postoperative VAS pain scores at 24 h	[2, 3, 6]	Compared with the TAPB, the bilateral ESPB is a more feasible and effective method for intra- and postoperative analgesia.
Jinaworn., et al. 2024 (23)	Thailand	31 vs.30	36.06 ± 9.41 vs. 35.80 ± 8.53	Metabolic bariatric surgery	ESPB	No block	Postoperative morphine consumption at 24 h via PCA	[1, 8, 9]	ESPB did not reduce morphine consumption or QoR.
Karaveli., et al. 2025 (24)	Turkey	20 vs. 20	40.05 ± 13.63 vs. 41.45 ± 12.41	Laparoscopic sleeve gastrectomy	US-guided bilateral ESPB	No block	Postoperative opioid consumption at 24 h	[1]	ESPB significantly reduced both intraoperative and postoperative analgesic consumptions and provided effective postoperative pain control.
Mostafa., et al. 2021 (14)	Egypt	30 vs. 30	38.80 ± 6.65 vs. 40.30 ± 7.86	Laparoscopic bariatric surgery	US-guided bilateral ESPB	Sham block (normal saline)	Postoperative pain scores	[1, 2]	ESPB provided satisfactory postoperative analgesia with decreased analgesic consumption without significant difference in postoperative pulmonary functions.
Toprak., et al. 2023 (25)	Turkey	40 vs.40	37.60 ± 9.87 vs. 37.50 ± 10.15	Bariatric surgery	US-guided bilateral ESPB	No block	Postoperative QoR-40 at 24 h	[1, 2, 3, 5, 6]	ESPB improved postoperative quality of recovery, reduced NRS scores, and total analgesic consumption.
ul Huda., et al. 2024 (26)	Kingdom of Saudi Arabia	25 vs.25	37.88 (34–45.5) vs. 35 (23.5–41.5)	Laparoscopic sleeve gastrectomy	US-guided bilateral ESPB	No block	Postoperative pain scores at 24 h	[2, 6]	ESPB is associated with a significant reduction in intraoperative and 24 h postoperative opioid consumption.
Wang., et al. 2023 (27)	China	76 vs. 75	32.8 ± 6.5 vs. 33.0 ± 6.4	Laparoscopic sleeve gastrectomy	US-guided bilateral ESPB	Sham block (normal saline)	Postoperative QoR-15 at 24 and 48 h	[1, 2, 4, 7, 10]	Single ESPB does not improve the global QoR-15 scores after laparoscopic sleeve gastrectomy, but the pain scores reduced.
Zengin., et al. 2021 (28)	Turkey	30 vs.30	40.2 ± 12.2 vs. 39.4 ± 10.5	Laparoscopic bariatric surgery	US-guided bilateral ESPB	LIA	Total intraoperative opioid consumption	[1, 3]	ESPB appears to be a simple and effective technique to improve perioperative pain control and reduce intraoperative opioid need.

ESPB, erector spinae plane block; QLB, quadratus lumborum block; US, ultrasound; PCA, patient-controlled analgesia; LIA, local infiltration analgesia. Secondary outcomes: 1. Pain scores (at rest or during movement at different time points); 2. Perioperative opioid consumption; 3. The first time need analgesics; 4. The numbers of need analgesics; 5. Time of first ambulation; 6. Adverse events (postoperative nausea, vomiting, itching, urine retention); 7. Regional blocks related complications; 8. Quality of recovery (15 or 40); 9. Patients satisfaction scores; and 10. Lengthy of hospital stay.

Among the included studies, two were designed as three-arm comparators (6, 7), while the remaining eight were two-arm comparators (13, 14, 23–28). Four RCTs compared erector spinae plane block (ESPB) with non-block care (23–26), two compared ESPB with sham block (14, 27), and the remaining four compared ESPB with other regional anesthesia techniques, including TAPB (6, 13), QLB (7), and local infiltration analgesia (LIA) (28), respectively (Table 2).

**Table 2:** Details of regional blocks and postoperative pain management.

Study	Punctures cite (ESPB vs. control)		Local anesthetics use (ESPB vs. control)		Time of block (ESPB vs. control)		Postoperative pain management
Abdelhamid., et al. 2020 (6)	T9	1. TAPB (subcostal) 2. No block	30 ml of 0.25% bupivacaine (15 mL for each side)	TAPB (30 mL of 0.25% bupivacaine on each side with supine position)	After GA	After GA	1. Paracetamol (with maximum daily dose of 4 g/24 h) 2. 50 mg of i.v pethidine
Ashoor., et al. 2023 (7)	T7	1. QLB 2. Sham block	30 mL of 0.25% bupivacaine (same technique was repeated on opposite site)	30 mL of 0.25% bupivacaine (same technique was repeated on opposite site)	Before extubation	Before extubation	1. PCA 2. 1 g of i.v paracetamol 3. 30 mg of ketorolac (not exceeding 120 mg/day)
Elshazly., et al. 2022 (13)	T5 (prone position)	TAPB	20 mL 0.25% bupivacaine on each side	20 mL 0.25% bupivacaine on each side	Before surgery	Before surgery	1. Nalbuphine 0.1 mg (a maximum dose of 50 mg/24 h) 2. Paracetamol (1 g/8 h), nalbuphine (0.1 mg/kg/8 h), ketorolac (0.5 mg/kg/6 h)
Jinaworn., et al. 2024 (23)	T7 (positioned in a seated posture)	No block	0.25% bupivacaine 25 mL one each side	NA	Before GA	NA	1. PCA 2. 1 g of i.v paracetamol and 40 mg of parecoxib 3. i.v fentanyl 25 μg every 15 min
Karaveli., et al. 2025 (24)	T7 (prone position)	No block	0.25% bupivacaine 20 mL one each side	NA	Before GA	NA	1. Paracetamol 1 g; i.v. at 8 h intervals 2. Tramadol 100 mg i. v
Mostafa., et al. 2021 (14)	T7	Sham block	0.25% bupivacaine 20 mL on each side	20 ml normal saline on each side	Before GA	Before GA	1. PCA 2. i. v morphine in 3 mg every 5 min
Toprak., et al. 2023 (25)	T7 (sitting position)	No block	20 mL of 0.25 bupivacaine on each side	NA	Before GA	NA	1. Paracetamol 1 g i.v. every 8 h and tenoxicam 20 mg i.v. every 12 h 2. 100 mg i.v. of tramadol
ul Huda., et al. 2024 (26)	T9 (lateral position)	No block	15 mL of 0.25% ropivacaine on each side	NA	After GA	NA	1. PCA 2. i.v 2 mg morphine
Wang., et al.2023 (27)	T7 (left lateral decubitus position)	Sham block	30 mL of 0.33% ropivacaine on each side	0.9% normal saline at the same dosage on each side	Before GA	Before GA	1. PCA 2. 40 mg parecoxib
Zengin., et al. 2021 (28)	T9 (sitting position)	LIA	20 mL 0.5% bupivacaine and 5 mL 0.2% lidocaine	5 mL 0.5% bupivacaine injection to each trocar site (total of 25 mL)	Before GA	Before surgery	1 g paracetamol, and the control group received 150 mg tramadol

ESPB, erector spinae plane block; PCA, patient-controlled analgesia; GA, general anesthesia; NA, not applicable; QLB, quadratus lumborum block; LIA, local infiltration analgesia.

### 3.2 Primary outcome

#### 3.2.1 Postoperative opioid consumption (morphine equivalent) at 24 h

**Figure 2:**
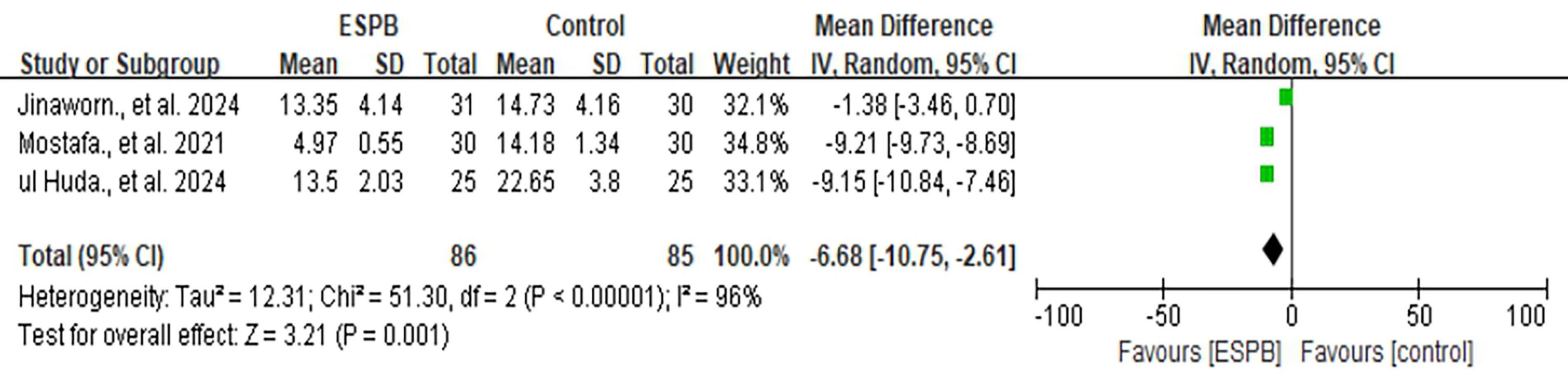
The forest plot of postoperative opioid consumption (morphine equivalent) at 24 h between ESPB and control group.

Three studies compared ESPB with control groups (non-block care or sham block) in terms of cumulative opioid consumption (intravenous morphine equivalents, mg) at 24 h postoperatively (Figure 2) (14, 23, 26). Pooled data from these RCTs demonstrated that ESPB significantly reduced postoperative opioid consumption compared to the control group [MD −6.68; 95% CI −10.75, −2.61; *p* = 0.001; I^2^ = 96%]. Due to the limited number of studies, subgroup analysis based on different regional anesthesia techniques was not performed. Sensitivity analysis, conducted by sequentially removing two studies (14, 26), identified the source of heterogeneity, which remained high (Supplementary Table 1).

### 3.3 Secondary outcomes

#### 3.3.1 Age and BMI

**Figure 3:**
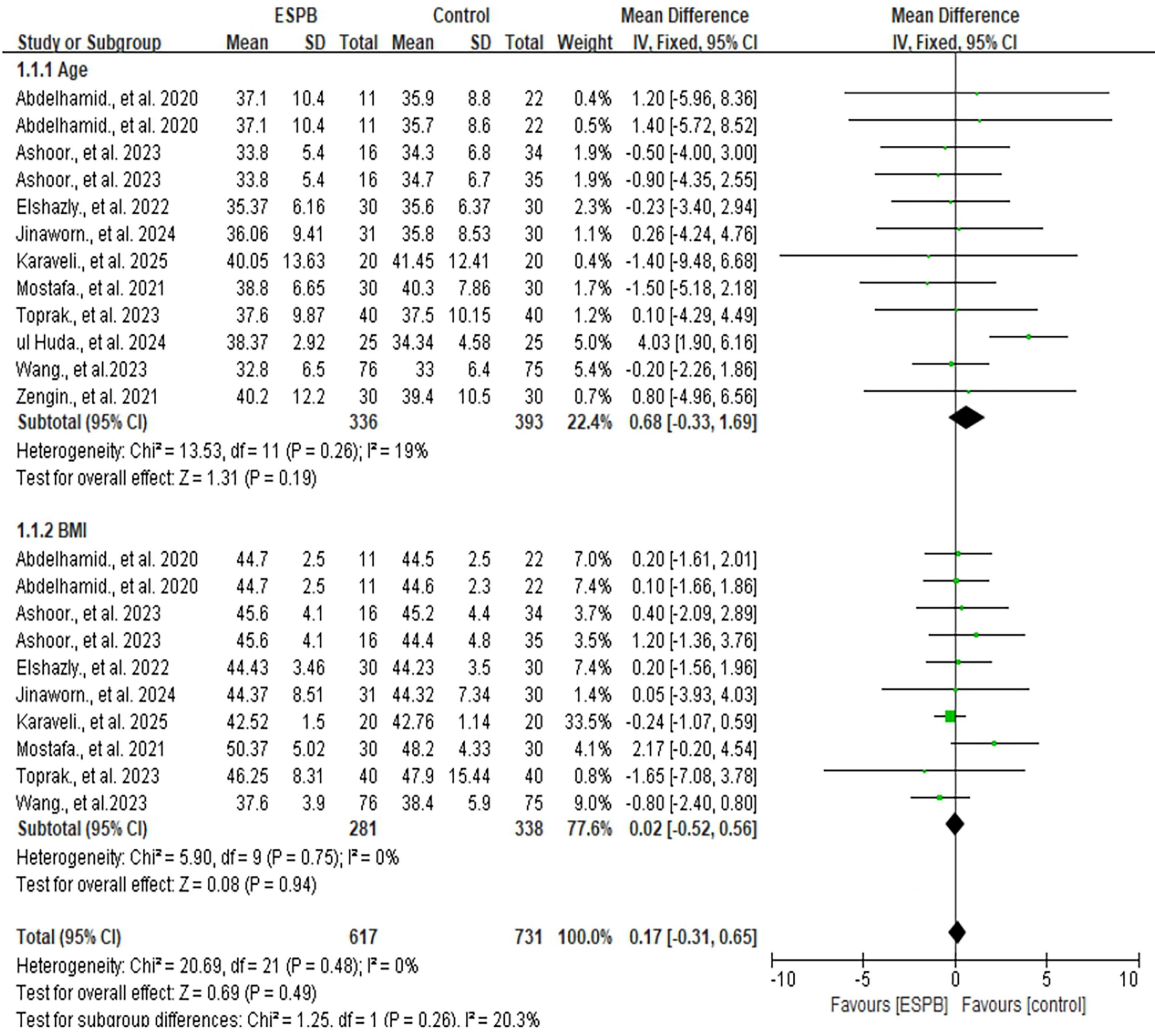
The forest plot of age and BMI between ESPB and control group.

All included studies reported data on age [MD 0.68; 95% CI −0.33, 1.69; *p* = 0.19; I^2^ = 19%] and BMI [MD 0.02; 95% CI −0.52, 0.56; *p* = 0.94; I^2^ = 0%] (6, 7, 13, 14, 23–28). No significant differences were observed between the ESPB and control groups for either variable (Figure 3). Considering the I^2^<50%, a fixed model was applied.

#### 3.3.2 Postoperative rest and movement pain scores at different time points

No significant differences were observed in postoperative rest pain scores at 0 h [MD −0.79; 95% CI −4.52, 2.94; *p* = 0.68; I^2^ = 70%], 30 min [MD −0.83; 95% CI −1.92, 0.25; *p* = 0.13; I^2^ = 100%], 1 h [MD −0.16; 95% CI −1.23, 0.90; *p* = 0.76; I^2^ = 62%], 2 h [MD −0.50; 95% CI −1.55, 0.55; *p* = 0.35; I^2^ = 100%], 6 h [MD −0.43; 95% CI −1.02, 0.16; *p* = 0.15; I^2^ = 77%], 12 h [MD −0.57; 95% CI −1.27, 0.14; *p* = 0.11; I^2^ = 80%], 18 h [MD −1.64; 95% CI −4.65, 1.36; *p* = 0.28; I^2^ = 79%], and 48 h [MD −0.31; 95% CI −1.01, 0.39; *p* = 0.38; I^2^ = 86%]. However, at 24 h, ESPB was associated with significantly lower rest pain scores compared to the control group [MD −0.78; 95% CI −1.10, −0.46; *p*<0.00001; I^2^ = 41%] in favor of ESPB compared with control group (Supplementary Figure 1).

Similarly, no significant differences were observed in movement pain scores at 0 h [MD −0.20; 95% CI −3.36, 2.96; *p* = 0.90; I^2^ = 50%], 30 min [MD −0.29; 95% CI −0.99, 0.41; *p* = 0.42; I^2^ = 78%], 1 h [MD −0.33; 95% CI −0.96, 0.30; *p* = 0.30; I^2^ = 11%], 2 h [MD −0.56; 95% CI −1.27, 0.16; *p* = 0.13; I^2^ = 83%], and 18 h [MD −1.89; 95% CI −5.44, 1.67; *p* = 0.30; I^2^ = 78%] also demonstrated that no significant difference between ESPB and control group. However, ESPB was associated with significantly lower movement pain scores at 6 h [MD −1.02; 95% CI −1.12, −0.92; *p*<0.00001; I^2^ = 0%], 12 h [MD −1.00; 95% CI −1.13, −0.87; *p*<0.0001; I^2^ = 42%], 24 h [MD −0.82; 95% CI −1.23, −0.42; *p*<0.00001; I^2^ = 0%], and 48 h [MD −0.80; 95% CI −1.07, −0.54; P<0.0001; I^2^ = 37%] respectively (Supplementary Figure 2).

#### 3.3.3 Duration of anesthesia and surgery time

Three studies compared the duration of anesthesia time between ESPB and control groups (ESPB vs. QLB, no-block care) (7, 23, 24), revealing no significant differences [MD −0.57; 95% CI −13.06, 11.92; *p* = 0.97; I^2^ = 97%]. Similarly, no significant differences were observed in surgery time [MD −1.04; 95% CI −4.11, 2.03; *p* = 0.51; I^2^ = 66%] (Supplementary Figure 3).

#### 3.3.4 Stay in PACU, the first time need analgesics, first ambulation time, and length of hospital stay

No significant differences were observed in PACU stay [MD −0.75; 95% CI −3.00, 1.49; *p* = 0.51; I^2^ = 95%] (7, 27), first ambulation [MD −0.41; 95% CI −1.30, 0.48; *p* = 0.36; I^2^ = 90%] (7, 25, 27), and lengthy of hospital stay [MD −0.16; 95% CI −0.55, 0.24; *p* = 0.44; I^2^ = 95%] (7, 24, 27) between ESPB and control group. However, patients receiving ESPB had a significantly prolonged time to first analgesic requirement [MD 14.17; 95% CI 5.50, 22.85; *p* = 0.001; I^2^ = 100%] (6, 7, 13, 14) (Supplementary Figure 4).

#### 3.3.5 PONV and patients’ satisfaction scores

Two studies compared the incidence of PONV between ESPB and control groups (24, 26) (ESPB vs. no block-care), revealing no significant differences [RR 0.77; 95% CI 0.39, 1.51; *p* = 0.45; I^2^ = 0%] (Supplementary Figure 5). Similarly, three studies found no significant differences in patient satisfaction scores between ESPB and control groups (7, 23, 24) (ESPB vs. QLB, no-block care) [MD 0.79; 95% CI −0.09, 1.67; *p* = 0.08; I^2^ = 97%] (Supplementary Figure 6).

#### 3.3.6 Postoperative QoR-15/40

One study reported higher QoR-15 scores at 24 h in the ESPB group compared to non-block care (175.02 ± 11.25 vs. 167.78 ± 18.59, *p* < 0.05) (25). However, another study comparing ESPB with sham block found no significant differences in QoR-40 scores at 24 h [115 (103–132) vs. 114 (101–126), *p* = 0.26] or 48 h [132 (110–144) vs. 129 (118–136), *p* = 0.22] found no significance different between two groups (27).

#### 3.3.7 Regional blocks related to complications

No procedure-related complications, such as nerve injury, pneumothorax, hematoma formation, or local anesthetic systemic toxicity, were reported in any of the included studies.

### 3.4 Publication bias

Due to the high heterogeneity (I^2^ > 50%) and limited number of included RCTs, publication bias was not assessed using funnel plots. Sensitivity analysis using the leave-one-out approach revealed no significant changes in the pooled effect size. The risk of bias was assessed using the Cochrane Risk of Bias Tool 2 (Supplementary Table 2). Using GRADE, the certainty of evidence for both primary and secondary outcomes ranged from moderate to high (Supplementary Table 3). Specifically, the key end-points—24 h opioid consumption, pain scores at rest and on movement, and time to first rescue analgesic—were all supported by moderate- to high-certainty evidence.

## 4 Discussion

This meta-analysis demonstrates that ultrasound-guided ESPB significantly reduces postoperative opioid consumption at 24 h compared to non-block care and sham block in patients undergoing metabolic surgery (*p* = 0.001). The observed 6.68 mg reduction in 24 h morphine consumption is clinically relevant. Although it falls short of the 10 mg threshold identified as the minimal clinically important difference after arthroplasty, this reduction remains meaningful in the context of metabolic surgery, where patients with obesity are particularly susceptible to opioid-related respiratory depression and other adverse events. Additionally, ESPB was associated with lower resting pain scores at 24 h (*p* < 0.00001) and reduced movement pain scores at 6, 12, 24, and 48 h postoperatively (*p* < 0.00001 for all time points). These findings suggest that ESPB provides effective postoperative analgesia, which is particularly relevant in metabolic surgery, where pain management is crucial for early mobilization and recovery. Furthermore, ESPB prolonged the time to first rescue analgesic requirement (*p* = 0.001), indicating its potential to reduce the need for additional opioid administration, which is beneficial in minimizing opioid-related side effects such as PONV. Interestingly, despite the analgesic benefits, ESPB did not significantly reduce the incidence of PONV or improve patient satisfaction scores (*p* = 0.45 and *p* = 0.08, respectively). This may be attributed to several factors. First, the limited number of studies reporting PONV outcomes could introduce bias (24, 26). Second, the postoperative pain management protocols in most studies included opioids and nonsteroidal anti-inflammatory drugs (NSAIDs), which are known to contribute to PONV. This highlights the need for future studies to explore multimodal analgesic regimens that minimize opioid use while maximizing the benefits of regional anesthesia techniques like ESPB (29). The safety profile of ESPB was notable, as none of the included studies reported complications such as nerve injury, pneumothorax, hematoma formation, or local anesthetic systemic toxicity (LAST). No complications were reported, but studies lacked systematic assessment (e.g., neurological exams, local anesthetic toxicity screens). Small samples and short follow-up preclude definitive safety conclusions. The safety of ESPB in obesity requires larger trials with protocolized monitoring. Notably, all included RCTs employed a bilateral single-injection technique, obviating the need for continuous catheterization and thereby reducing the risk of catheter-related complications and enhancing the practical applicability of ESPB in routine metabolic surgical care.

Four studies compared ESPB with no block (23–26), yielding conflicting results. Reported that ESPB did not significantly reduce morphine consumption or improve quality of recovery (23). In contrast, the other three studies demonstrated that ESPB significantly reduced both intraoperative and postoperative analgesic consumption, provided effective postoperative pain control, and enhanced postoperative quality of recovery (24–26). Similarly, two studies comparing ESPB with sham block found that ESPB offered satisfactory postoperative analgesia and reduced analgesic consumption (14). However, while ESPB effectively lowered pain scores, it did not improve global QoR-15 scores (27). When compared to other regional anesthesia techniques, such as TAPB and QLB, ESPB provided comparable or superior pain control, as evidenced by reduced pain scores and opioid consumption in patients undergoing metabolic surgery (6, 7, 13, 28). The variability in clinical outcomes across studies may also be influenced by factors such as patient positioning during block administration, injection speed, local anesthetic volume, and comparator type. Recent imaging studies have shown that prone positioning and higher injection speeds can lead to wider spread of local anesthetics, potentially enhancing the analgesic efficacy (30). These factors should be considered when designing future trials to optimize ESPB protocols (8). Thus, these reasons also may contribute to the different clinical results. In the current study, seven studies recorded the ESPB injection positions (13, 23–28) (prone position, seated posture, and lateral position). Moreover, most of included studies used 30 mL of 0.25% bupivacaine 15 to 30 mL on each side before or after general anesthesia induction. In addition, performing ESPB in obesity may also pose several challenges. First, deep fascial planes (>4–6 cm) limit ultrasound penetration, requiring low-frequency curvilinear probes (10). Second, prone positioning may be impractical; lateral decubitus optimizes ergonomics. Third, targeting T7–T9 achieves optimal dermatomal coverage for upper abdominal surgery (11). Last but not least, heterogeneity may also arise from center-specific expertise or equipment. Future studies should standardize those imaging protocols.

Several study limitations should be considered when interpreting our results: First, this study only included 10 RCTs and included moderate samples. The lack of quantity analysis did not assess publication bias by Egger or Begg regression. Second, the results of this study showed ESPB may not have a promising analgesia effect compared with non-block care, TAPB, QLB, and LIA in patients undergoing metabolic surgery. Third, all the included studies only described different metabolic surgeries but did not classify the types of incisions and surgical techniques involved, so selection bias may underestimate the analgesic efficacy of the ESPB. Last but not the least, heterogeneity stems from methodological diversity, including control types (sham vs. no-block), anesthetic volumes (15–30 mL), and administration timing. Most of the literature is composed of small and moderate sample sizes, with the largest included experimental group including 151 patients. Future studies should perform more analyses of ESPB, which would provide great incentive for setting guidelines for perioperative pain management.

## 5 Conclusion

In summary, this meta-analysis offers moderate evidence that ultrasound-guided ESPB constitutes an effective and safe analgesic strategy for patients undergoing metabolic surgery. ESPB significantly reduces postoperative opioid consumption and pain scores, particularly at 24 h postoperatively, while prolonging the time to first rescue analgesic requirement. Although ESPB did not significantly reduce PONV or improve patient satisfaction scores in this analysis, its favorable safety and ease of performance make it a valuable addition to multimodal analgesic regimens in metabolic surgery.

Future research should focus on standardizing ESPB techniques and exploring its role in multimodal analgesia to further minimize opioid use and enhance recovery outcomes. Additionally, larger, well-designed RCTs are needed to evaluate the impact of ESPB on patient-reported outcomes, such as quality of recovery and satisfaction, as well as its comparative effectiveness against other regional anesthesia techniques. Until then, ESPB should be considered a promising option for postoperative pain management in metabolic surgery, particularly in settings where opioid-sparing strategies are prioritized.

## Data Availability

The original contributions presented in the study are included in the article/Supplementary material, further inquiries can be directed to the corresponding author.
