# Supplementary material for: Analgesic efficacy of ultrasound-guided ESPB on metabolic surgery
Source: Front Med (Lausanne). 2025 Sep 2;12:1630657. doi: 10.3389/fmed.2025.1630657 (PMC12436430; doi:10.3389/fmed.2025.1630657)
Supplement: Supplementary file 1 [file Table_1.doc]

Supplementary Table 1. sensitivity analysis of postoperative opioid consumption (morphine equivalent) at 24h between ESPB and control group.

| Study | Z effect | MD | 95%CI | I^2^ | P value |
| --- | --- | --- | --- | --- | --- |
| Jinaworn., et al. 2024 | 36.41 | -9.20 | -9.70, -8.71 | 0% | ﹤0.0001 |
| Mostafa., et al. 2021 | 1.36 | -5.29 | -12.29, 2.23 | 97% | 0.17 |
| ul Huda., et al. 2024 | 1.37 | -5.36 | -13.03, 2.31 | 98% | 0.17 |

Supplementary Table 2. Risk bias of assessment of included studies.

| Study | Bias arising from the randomization process | Bias due to deviations from intended interventions | Bias due to missing outcome data | Bias in measurement of the outcome | Bias in selecting the reported result | Overall bias |
| --- | --- | --- | --- | --- | --- | --- |
| Abdelhamid., et al. 2020 | N | N | N | N | N | Low risk of bias |
| Ashoor., et al. 2023 | N | N | N | Y | N | Some concerns |
| Elshazly., et al. 2022 | N | N | N | N | N | Low risk of bias |
| Jinaworn., et al. 2024 | PY | N | N | Y | N | Some concerns |
| Karaveli., et al. 2025 | N | N | N | N | N | Low risk of bias |
| Mostafa., et al. 2021 | N | N | N | Y | N | Some concerns |
| Toprak., et al. 2023 | N | N | N | Y | N | Some concerns |
| ul Huda., et al. 2024 | N | N | N | Y | N | Some concerns |
| Wang., et al.2023 | N | N | N | N | N | Low risk of bias |
| Zengin., et al. 2021 | N | N | N | Y | N | Some concerns |

Supplementary Table 3. Quality of evidence assessment for main results.

| Study | ESPB *vs.* control | Corresponding risk of ESPB in resting pain score  *vs.* control | Certainty |
| --- | --- | --- | --- |
| Primary outcome | | | |
| Postoperative opioid consumption at 24h | 86 *vs.*85 | -6.68(-10.75, -2.61) | ⨁⨁⨁⨁ High |
| Secondary outcomes | | | |
| Age | 336 *vs.* 393 | 0.68(-0.33,1.69) | ⨁⨁⨁⨁ High |
| BMI | 281 *vs.*338 | 0.02(-0.52,0.56) | ⨁⨁⨁⨁ High |
| Resting pain scores at 0h | 51 *vs.*50 | -0.79(-4.52, 2.94) | ⨁⨁⨁◯ Moderate |
| Resting pain scores at 30min | 148 *vs.* 184 | -0.83(-1.92,0.25) | ⨁⨁⨁◯ Moderate |
| Resting pain scores at 1h | 91 *vs.* 90 | -0.16(-1.23, 0.90) | ⨁⨁⨁◯ Moderate |
| Resting pain scores at 2h | 148 *vs.*184 | -0.5(-1.55, 0.55) | ⨁⨁⨁◯ Moderate |
| Resting pain scores at 6h | 167 *vs.* 165 | -0.43(-1.02, 0.16) | ⨁⨁⨁◯ Moderate |
| Resting pain scores at 12h | 167 *vs.* 165 | -0.57(-1.27, 0.14) | ⨁⨁⨁◯ Moderate |
| Resting pain scores at 18h | 51 *vs.*50 | -1.64(4.65, 1.36) | ⨁⨁⨁◯ Moderate |
| Resting pain scores at 24h | 167 *vs.* 165 | -0.78(-1.1, -0.46) | ⨁⨁⨁◯ Moderate |
| Resting pain scores at 48h | 116 *vs.* 115 | -0.31(-1.01, 0.39) | ⨁⨁⨁◯ Moderate |
| Movement pain scores at 0h | 51 *vs.*50 | -0.2(-3.36, 2.96) | ⨁⨁⨁◯ Moderate |
| Movement pain score at 30min | 116 *vs.* 115 | -0.29(-0.99, 0.41) | ⨁⨁⨁◯ Moderate |
| Movement pain scores at 1h | 91 *vs.* 90 | -0.33(-0.96, 0.3) | ⨁⨁⨁◯ Moderate |
| Movement pain scores at 2h | 116 *vs.* 115 | -0.56(-1.27, 0.16) | ⨁⨁⨁◯ Moderate |
| Movement pain scores at 6h | 167 *vs.*165 | -1.02(-1.12, -0.92) | ⨁⨁⨁◯ Moderate |
| Movement pain scores at 12h | 167 *vs.*165 | -1(-1.13, -0.87) | ⨁⨁⨁◯ Moderate |
| Movement pain scores at 18h | 51 *vs.*50 | -1.89(-5.44, 1.67) | ⨁⨁⨁◯ Moderate |
| Movement pain scores at 24h | 167 *vs.*165 | -0.82(-1.23, -0.42) | ⨁⨁⨁◯ Moderate |
| Movement pain scores at 48h | 116 *vs.* 115 | -0.8(-1.07, -0.54) | ⨁⨁⨁◯ Moderate |
| Surgery time | 83 *vs.* 119 | -0.57(-13.06, 11.92) | ⨁⨁⨁⨁ High |
| Anesthesia time | 236 vs. 293 | -1.04(-4.11, 2.03) | ⨁⨁⨁⨁ High |
| PACU time | 108 *vs.*144 | -0.75(-3, 1.49) | ⨁⨁⨁⨁ High |
| The first-time need of analgesics | 114 *vs.*173 | 14.17(5.5, 22.85) | ⨁⨁⨁⨁ High |
| First ambulation time | 148 *vs.* 184 | -0.41(-1.3, 0.48) | ⨁⨁⨁⨁ High |
| Length of hospital stay | 128 *vs.* 164 | -0.16(-0.55, 0.24) | ⨁⨁⨁⨁ High |
| PONV | 10/45(22.2%) *vs.*13/45(28.9%) | 0.77(0.39, 1.51) | ⨁⨁⨁⨁ High |
| Patients’ satisfaction scores | 83 *vs.*119 | 0.79(-0.09, 1.67) | ⨁⨁⨁⨁ High |
