# Supplementary material for: Analgesic efficacy of ultrasound-guided ESPB on metabolic surgery
Source: Front Med (Lausanne). 2025 Sep 2;12:1630657. doi: 10.3389/fmed.2025.1630657 (PMC12436430; doi:10.3389/fmed.2025.1630657)
Supplement: Supplementary file 2 [file Table_2.doc]

Pubmed: 2025-02-24,18

| #1 **(((((("Bariatric Surgery"[Mesh]) OR (laparoscopic bariatric surgery[Title/Abstract])) OR (Bariatric Surgeries[Title/Abstract])) OR (Metabolic Surgery[Title/Abstract])) OR (Metabolic Surgeries[Title/Abstract])) OR (Bariatric Surgical Procedures[Title/Abstract])) OR (Bariatric Surgical Procedure[Title/Abstract])** |
| --- |
| #2 **(erector spinae plane block[Title/Abstract]) OR (erector spinae block[Title/Abstract])** |
| #3 #1 AND #2 |

Web of science:2025-02-24,21

| #1 ((((((TS=(Bariatric Surgery)) OR TS=(laparoscopic bariatric surgery)) OR TS=(Bariatric Surgeries)) OR TS=(Metabolic Surgery)) OR TS=(Metabolic Surgeries)) OR TS=(Bariatric Surgical Procedures)) OR TS=(Bariatric Surgical Procedure) |
| --- |
| #2 (TS=(erector spinae plane block)) OR TS=(erector spinae block) |
| #3 #1 AND #2 |

Cochrane library:2025-02-24,46

| #1 (erector spinae plane block):ti,ab,kw |
| --- |
| #2 (Bariatric Surgery):ti,ab,kw or (laparoscopic bariatric surgery):ti,ab,kw or (Metabolic Surgery):ti,ab,kw |
| #3 #1 and #2 |

Embase: 2025-02-24, 26

| #1 'erector spinae plane block' |
| --- |
| #2 'bariatric surgery' or 'laparoscopic bariatric surgery' or 'metabolic surgery' |
| #3 #1 and #2 |
