# Supplementary material for: Analgesic efficacy of ultrasound-guided ESPB on metabolic surgery
Source: Front Med (Lausanne). 2025 Sep 2;12:1630657. doi: 10.3389/fmed.2025.1630657 (PMC12436430; doi:10.3389/fmed.2025.1630657)

Supplementary Figure1. The forest plot of postoperative rest pain scores at different time points.


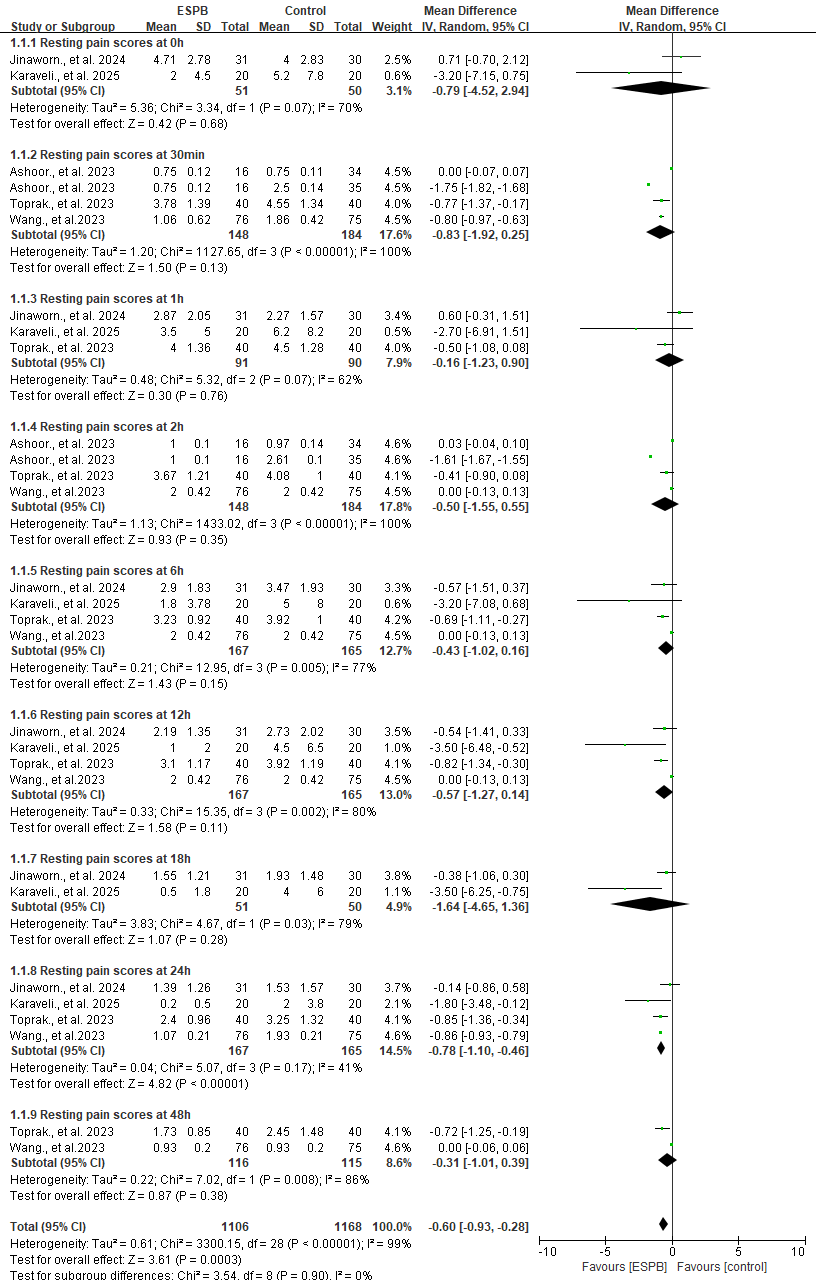


Supplementary Figure 2. The forest plot of postoperative movement pain scores at different time points.


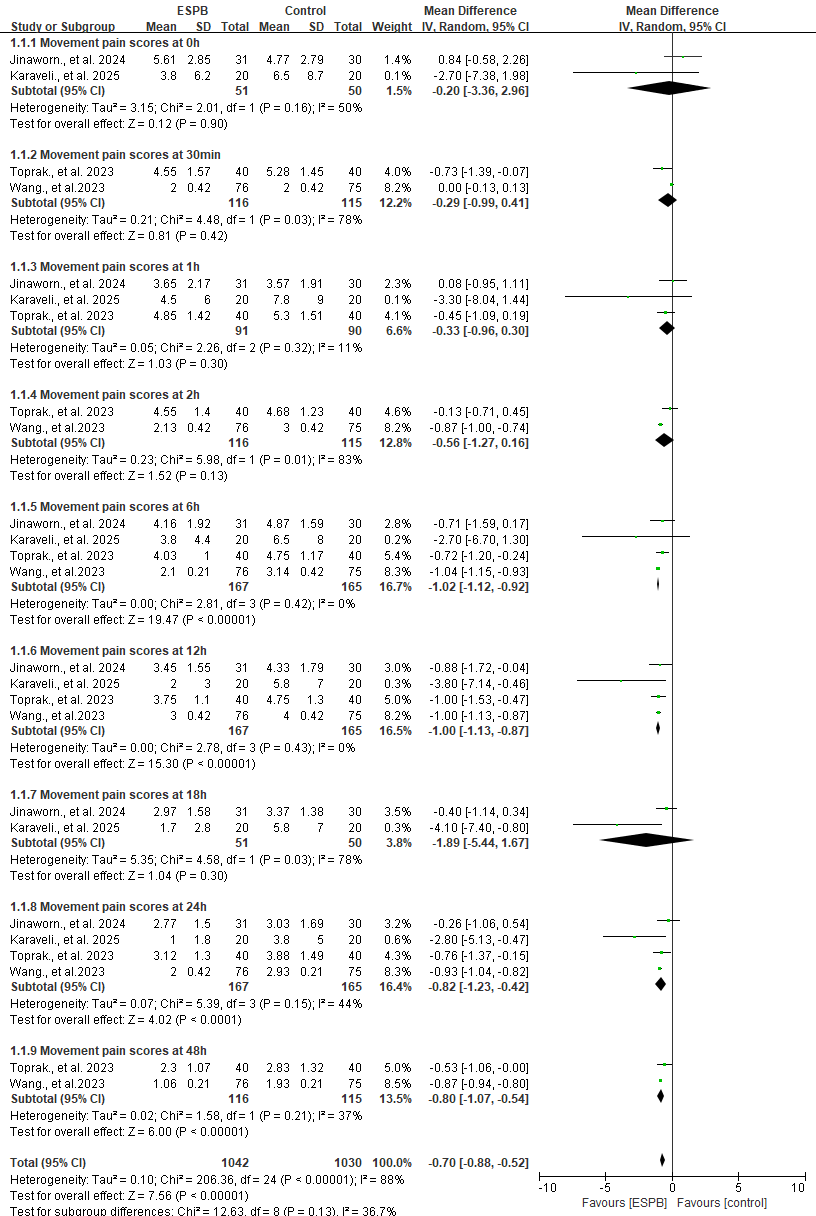


Supplementary Figure 3. The forest plot of anesthesia and surgery time between ESPB and control group.


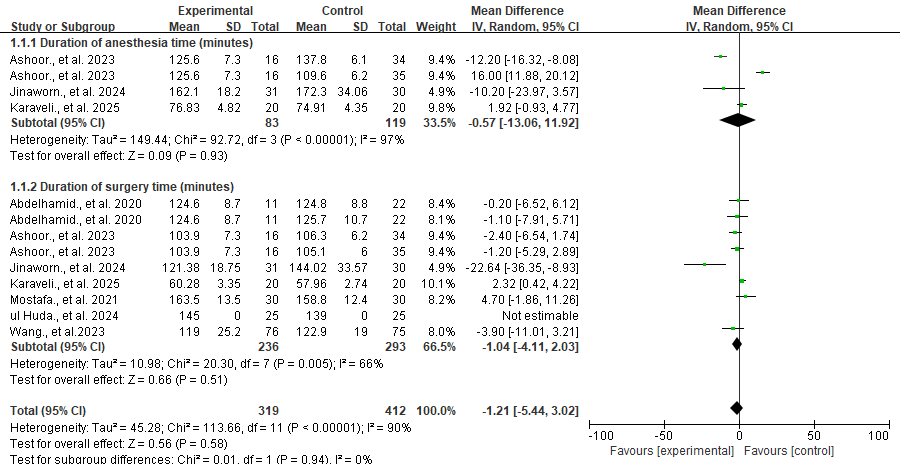


Supplementary Figure 4. The forest plot of stay in PACU, the first time need analgesics, first ambulation time, and length of hospital stay.


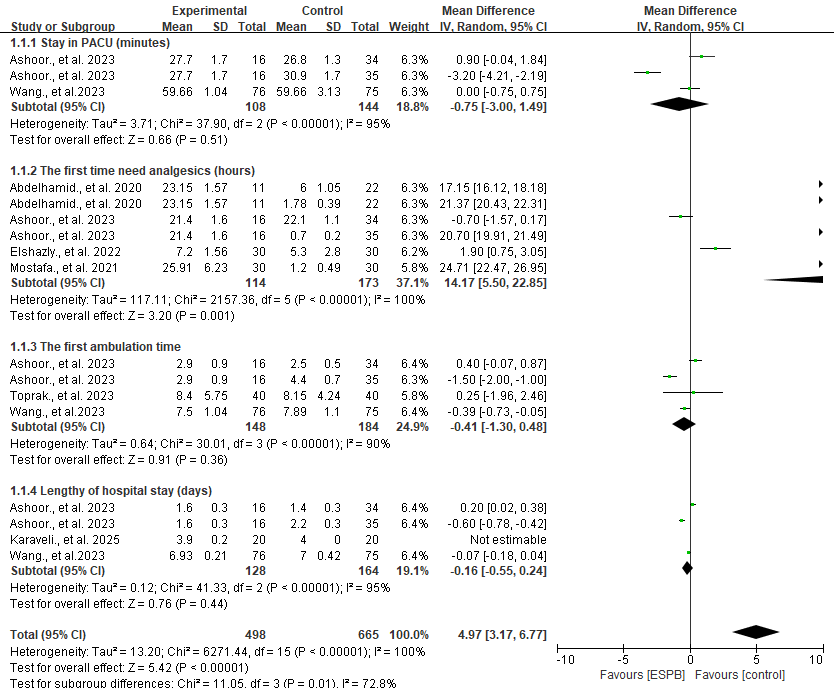


Supplementary Figure 5. The forest plot of stay in postoperative nausea and vomiting between ESPB and control group.


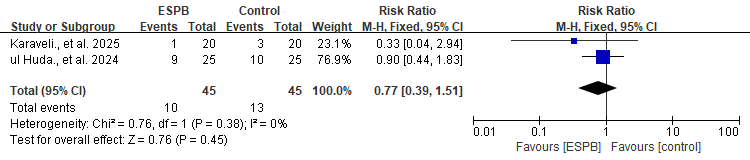


Supplementary Figure 6. The forest plot of stay in patients’ satisfaction scores.


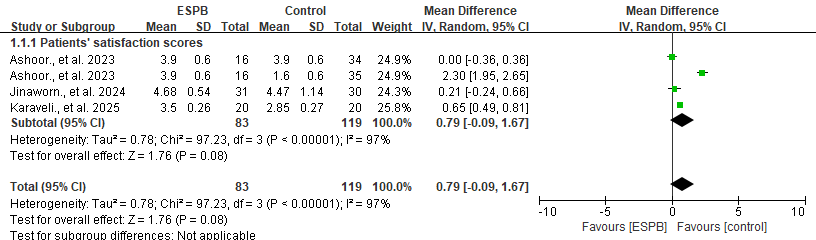

Supplement: Supplementary file 3 [file Data_Sheet_1.doc]
